# Effect of tranexamic acid on intracranial haemorrhage and infarction in patients with traumatic brain injury: a pre-planned substudy in a sample of CRASH-3 trial patients

**DOI:** 10.1136/emermed-2020-210424

**Published:** 2020-12-01

**Authors:** Abda Mahmood, Kelly Needham, Haleema Shakur-Still, Tim Harris, Sabariah Faizah Jamaluddin, David Davies, Antonio Belli, Fatahul Laham Mohamed, Caroline Leech, Hamzah Mohd Lotfi, Phil Moss, Fiona Lecky, Philip Hopkins, Darin Wong, Adrian Boyle, Mark Wilson, Melanie Darwent, Ian Roberts

**Affiliations:** 1 Clinical Trials Unit, London School of Hygiene & Tropical Medicine Faculty of Epidemiology and Population Health, London, UK; 2 Department of Emergency Medicine, Royal London Hospital, Barts Health NHS Trust, London, UK; 3 Emergency Department, Hospital Sungai Buloh, Sungai Buloh, Malaysia; 4 NIHR Surgical Reconstruction and Microbiology Research Centre, University Hospitals Birmingham NHS Foundation Trust, Birmingham, UK; 5 Emergency Department, Hospital Sultanah Bahiyah, Alor Setar, Malaysia; 6 Emergency Department, University Hospitals Coventry & Warwickshire NHS Trust, Coventry, UK; 7 Emergency Department, Hospital Sultanah Nur Zahirah, Kuala Terengganu, Malaysia; 8 Clinical Research Unit, Emergency Department, Saint George's University Hospitals NHS Foundation Trust, London, UK; 9 Accident & Emergency, Salford Royal NHS Foundation Trust, Salford, UK; 10 Emergency Department, King's College Hospital NHS Foundation Trust, London, UK; 11 Emergency Department, Penang General Hospital, Georgetown, Malaysia; 12 Emergency Department, Addenbrooke’s Hospital Cambridge University Hospitals NHS Foundation Trust, Cambridge, UK; 13 Neurosurgeries, Emergencies & Trauma, Division of Medicine, St Mary's Hospital, Imperial College Healthcare NHS Trust, London, UK; 14 Emergency Department, Oxford University Hospitals NHS Foundation Trust, Oxford, UK

**Keywords:** trauma, head, imaging, CT/MRI, emergency department, epidemiology, research, clinical

## Abstract

**Background:**

Early tranexamic acid (TXA) treatment reduces head injury deaths after traumatic brain injury (TBI). We used brain scans that were acquired as part of the routine clinical practice during the CRASH-3 trial (before unblinding) to examine the mechanism of action of TXA in TBI. Specifically, we explored the potential effects of TXA on intracranial haemorrhage and infarction.

**Methods:**

This is a prospective substudy nested within the CRASH-3 trial, a randomised placebo-controlled trial of TXA (loading dose 1 g over 10 min, then 1 g infusion over 8 hours) in patients with isolated head injury. CRASH-3 trial patients were recruited between July 2012 and January 2019. Participants in the current substudy were a subset of trial patients enrolled at 10 hospitals in the UK and 4 in Malaysia, who had at least one CT head scan performed as part of the routine clinical practice within 28 days of randomisation. The primary outcome was the volume of intraparenchymal haemorrhage (ie, contusion) measured on a CT scan done after randomisation. Secondary outcomes were progressive intracranial haemorrhage (post-randomisation CT shows >25% of volume seen on pre-randomisation CT), new intracranial haemorrhage (any haemorrhage seen on post-randomisation CT but not on pre-randomisation CT), cerebral infarction (any infarction seen on any type of brain scan done post-randomisation, excluding infarction seen pre-randomisation) and intracranial haemorrhage volume (intraparenchymal + intraventricular + subdural + epidural) in those who underwent neurosurgical haemorrhage evacuation. We planned to conduct sensitivity analyses excluding patients who were severely injured at baseline. Dichotomous outcomes were analysed using relative risks (RR) or hazard ratios (HR), and continuous outcomes using a linear mixed model.

**Results:**

1767 patients were included in this substudy. One-third of the patients had a baseline GCS (Glasgow Coma Score) of 3 (n=579) and 24% had unilateral or bilateral unreactive pupils. 46% of patients were scanned pre-randomisation and post-randomisation (n=812/1767), 19% were scanned only pre-randomisation (n=341/1767) and 35% were scanned only post-randomisation (n=614/1767). In all patients, there was no evidence that TXA prevents intraparenchymal haemorrhage expansion (estimate=1.09, 95% CI 0.81 to 1.45) or intracranial haemorrhage expansion in patients who underwent neurosurgical haemorrhage evacuation (n=363) (estimate=0.79, 95% CI 0.57 to 1.11). In patients scanned pre-randomisation and post-randomisation (n=812), there was no evidence that TXA reduces progressive haemorrhage (adjusted RR=0.91, 95% CI 0.74 to 1.13) and new haemorrhage (adjusted RR=0.85, 95% CI 0.72 to 1.01). When patients with unreactive pupils at baseline were excluded, there was evidence that TXA prevents new haemorrhage (adjusted RR=0.80, 95% CI 0.66 to 0.98). In patients scanned post-randomisation (n=1431), there was no evidence of an increase in infarction with TXA (adjusted HR=1.28, 95% CI 0.93 to 1.76). A larger proportion of patients without (vs with) a post-randomisation scan died from head injury (38% vs 19%: RR=1.97, 95% CI 1.66 to 2.34, p<0.0001).

**Conclusion:**

TXA may prevent new haemorrhage in patients with reactive pupils at baseline. This is consistent with the results of the CRASH-3 trial which found that TXA reduced head injury death in patients with at least one reactive pupil at baseline. However, the large number of patients without post-randomisation scans and the possibility that the availability of scan data depends on whether a patient received TXA, challenges the validity of inferences made using routinely collected scan data. This study highlights the limitations of using routinely collected scan data to examine the effects of TBI treatments.

**Trial registration number:**

ISRCTN15088122.

**Key messages**

**What is already known on this subject**

- The CRASH-3 trial showed that tranexamic acid (TXA) administered within 3 hours of injury reduced early head injury deaths in patients not at or close to the point of death. TXA treatment may have prevented deaths from post-traumatic intracranial bleeding. However, there is little evidence on the effect of TXA on intracranial bleeding as measured on CT head scans of patients with traumatic brain injury (TBI). Prior to this study, a meta-analysis of two randomised trials of TXA in TBI showed a reduction in intracranial bleeding with TXA, on CT scans that were mandated after randomisation. But because the trials were small (n=249, n=229), the results were inconclusive.

**What this study adds**

- This substudy in 1767 CRASH-3 trial patients found that TXA may reduce intracranial bleeding, after patients with unreactive pupils at baseline were excluded. This study improves knowledge of the strengths and limitations of using routine scan data to examine the effects of a clinical trial intervention in TBI.

## Introduction

The CRASH-3 trial was a randomised placebo-controlled trial of the effects of tranexamic acid (TXA) on death and disability in patients with traumatic brain injury (TBI).^1^ The results showed that TXA (1 g over 10 min followed by 1 g over 8 hours) administered within 3 hours of injury reduces early head injury deaths in non-moribund patients (ie, not at or close to the point of death) (relative risk (RR) 0·74, 95% CI 0·58 to 0·94).^2 3^ While the trial was underway, the data monitoring committee asked for additional CT scan data to be collected ‘to explore if, why and how patients are affected by TXA’. Consequently, we developed a separate substudy protocol to explore this aim.^4^ While the CRASH-3 trial was ongoing and before the results were unblinded, we examined brain scans that were acquired as part of routine clinical practice from a proportion of CRASH-3 trial patients at selected trial sites. Most TBI patients presenting to hospital have CT head scans as part of their routine clinical care.^5^

In this substudy, we used routinely collected scans to examine the mechanism of action of TXA in TBI, particularly the effects of TXA on intracranial bleeding and infarction.^4 6^ If patients who receive TXA have less intracranial bleeding or more cerebral infarction on their scans compared with those who receive placebo, this information, along with the results of the main CRASH-3 trial, could improve understanding of the mechanism of action of TXA and help generalise the CRASH-3 trial results.^7^

## Methods

The background,^8^ protocol^1^ and results,^2 3^ of the CRASH-3 trial have been reported previously. Adults with head injury who were within 3 hours of injury, and had a baseline Glasgow Coma Score (GCS) of ≤12 or any intracranial bleeding on CT, and no significant extracranial bleeding were eligible for randomisation in hospital. The time window for eligibility was originally 8 hours, but in 2016 the protocol was changed to limit recruitment to within 3 hours of injury. Patients with TBI were randomly allocated to receive TXA (loading dose 1 g over 10 min, then 1 g infusion over 8 hours) or placebo (0.9% sodium chloride), as an additional treatment to the standard management of TBI.

Patients, care givers and those assessing outcomes were masked to allocation.

Between July 2012 and January 2019, a total of 12 737 patients were randomised into the CRASH-3 trial in 175 hospitals across 29 countries, of whom 9202 patients were randomised within 3 hours of injury. This exploratory substudy (NCT01402882) was developed during the course of the CRASH-3 trial and the protocol and analysis plan were registered and published before the CRASH-3 trial was unblinded.^4 6^

**Figure 1:**
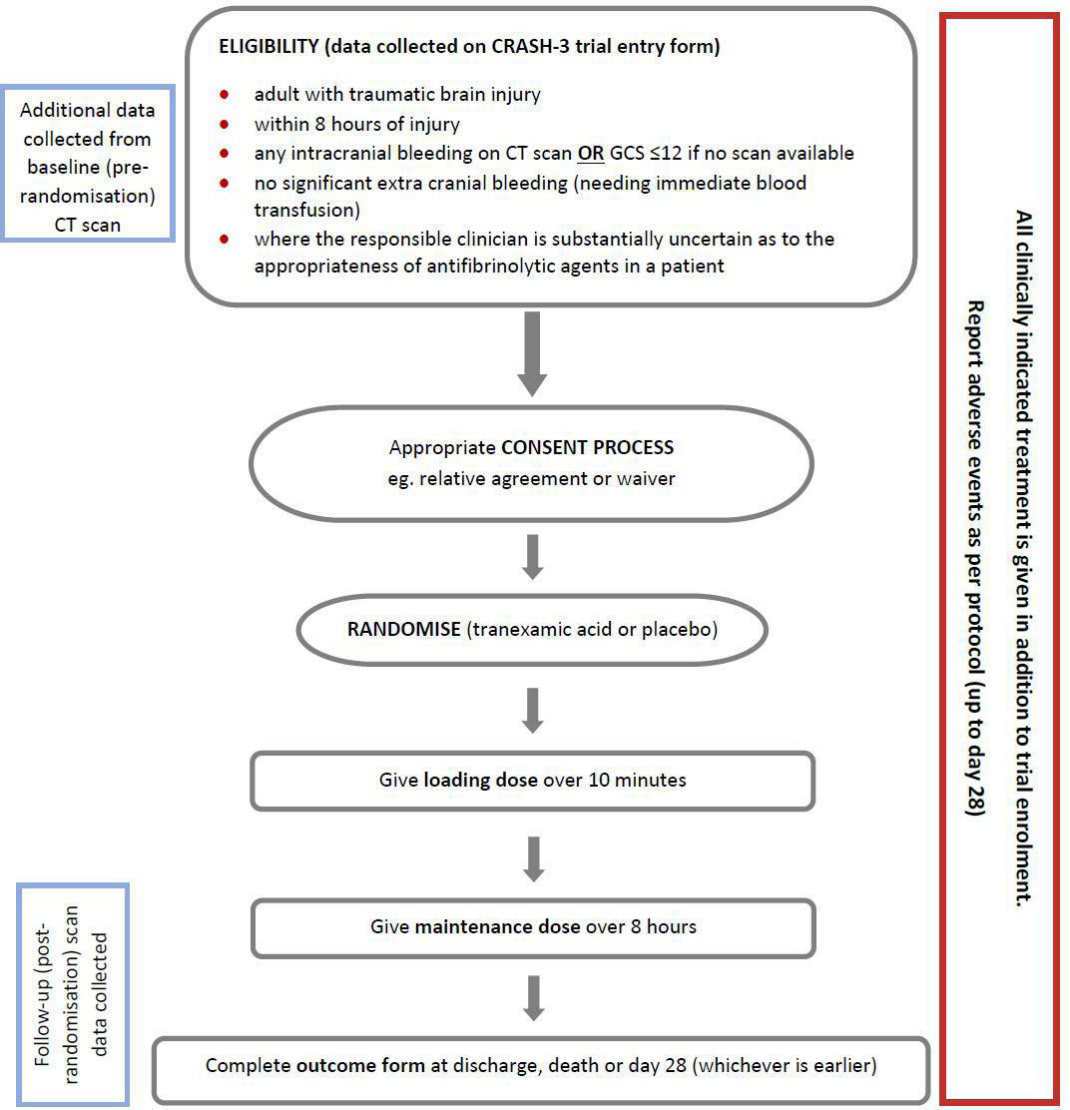
Flowchart: inclusion criteria for the CRASH-3 trial (blue boxes show additional procedure for the substudy). GCS, Glasgow Coma Score.

Patients who fulfilled the eligibility criteria for the CRASH-3 trial were eligible for this substudy (figure 1). While this substudy was underway, we restricted its eligibility to patients with GCS≤12 since many patients with mild TBI did not have a post-randomisation scan. In addition, patients needed to have had at least one CT scan within 28 days of randomisation, and be randomised before or during the time that the single assessor of the scans was on site.

### Sample size

The CRASH-2 trial substudy in 249 patients with head injury and extracranial bleeding who had one CT head scan done before randomisation and another mandated after randomisation found a 15% reduction in the volume of intracranial bleeding with TXA (24 mL TXA and 28 mL placebo), a correlation of 0.6 between pre-randomisation and post-randomisation bleeding volumes and a SD of 28 mL.^9^ We used this data from the CRASH-2 substudy to estimate an unadjusted sample size of 1542 patients to achieve 80% power to detect the expected treatment effect, which was then reduced to 987 patients with adjustment for pre-randomisation intracranial bleeding volume (1542×(1-(0.6^2^)). We originally planned for this substudy to be conducted in 1000 trial patients, but as data collection was underway, we increased the sample size because: (1) Many patients were recruited without a pre-randomisation CT scan, and so baseline adjustment would not improve power to the extent that we expected. (2) Many patients had their post-randomisation scan done so soon after randomisation that any effect of TXA would not have sufficient opportunity to manifest on these scans, and their inclusion might dilute the treatment effect. (3) Cerebral infarction occurred less frequently than intracranial bleeding so we would not be able to reliably examine the effects of TXA on infarction. The scan assessor had time to collect data from a maximum of 2000 patients before the CRASH-3 trial completed recruitment and so this was the sample size we aimed for.

### Participating hospitals

We invited CRASH-3 trial hospitals with high patient recruitment to take part in this substudy. The UK and Malaysia were two of the highest recruiting countries in the CRASH-3 trial and stored the brain images that were collected as part of the routine clinical practice on electronic systems that could be accessed in-hospital by the substudy assessor. We invited the highest recruiting hospitals from these countries to have the routinely collected brain scans of CRASH-3 trial patients examined for the purpose of this substudy. If it was possible for the substudy assessor to perform an on-site examination of the electronic brain imaging done as part of routine care at that hospital site, and the trial principal investigator at the hospital site was willing to take part, the assessor visited the site and this substudy was conducted at that site. A list of participating hospitals is shown in online supplemental appendix 1. All regulatory, ethical and local approvals were in place before data collection began.

10.1136/emermed-2020-210424.supp1 Supplementary data

### Outcome measurement

Non-contrast enhanced CT head scans that were done pre-randomisation and/or post-randomisation were examined. One outcome assessor (AM) who was blind to treatment allocation rated all scans in electronic (axial) format at each hospital using the local systems (ie, Picture Archiving and Communication System). The scans and neuroradiology reports were examined and rated using a standardised data collection form (online supplemental appendix 2).

10.1136/emermed-2020-210424.supp2 Supplementary data

To assess intra-rater reliability, a sample of 90 patients’ scans were rated on two occasions by the same assessor, with the second reading done blind to the first. In this case, the assessor attended the same hospital site on two occasions to re-examine this sample of scans.

The CRASH-3 trial entry forms were completed by clinical research staff in participating hospitals and included a question on the type of haemorrhage seen on baseline CT.^1^ To examine inter-rater reliability, these CRASH-3 trial entry data that were completed by clinical research staff in participating hospitals were compared against the pre-randomisation scan readings done by the substudy outcome assessor. The second reading (substudy outcome assessor) was done blind to the first (CRASH-3 trial entry form assessors).

The ABC/2 method, a validated scale, was used to estimate the volume of epidural haemorrhage (EDH), intraparenchymal haemorrhage (IPH) (ie, contusion) and intraventricular haemorrhage (IVH). The assessor selected the slice on which each haemorrhage was most visible. A point in the centre of the haemorrhage was found and two measurements taken: (A) the maximal diameter; (B) the width perpendicular to A. For the measurement of depth, the maximum number of slices on which the haemorrhage is visible is multiplied by slice thickness (C). These three measurements (in cm) are multiplied together and then divided by 2 to estimate the volume in cubic centimetres (ie, ml). This method assumes haemorrhage has an almost spherical shape and shows good agreement with automated volumetric analyses in IPH and EDH.^10 11^

The maximum thickness of subdural haemorrhage (SDH) was measured and this value substituted into a novel formula to estimate volume (see online supplemental appendix 3). This formula was developed by substudy collaborators at University Hospitals Birmingham (UK).^12^ It takes the difference between two spheres to represent the subdural space and divides this by 8 because the measurement is for unilateral SDH (divided by 2), which is typically thicker at the centre (divided by 2) and bound by superior-inferior and anterior-posterior cerebral axes (divided by 4).

10.1136/emermed-2020-210424.supp3 Supplementary data

The volume of subarachnoid haemorrhage (SAH) cannot be estimated, and so we recorded whether or not any SAH was visible (yes/no).

### Primary outcome

The primary outcome was the volume of IPH (ie, contusions) seen on the post-randomisation scan, adjusted for baseline covariates: time from injury to pre-randomisation scan, GCS score, pupil reaction, systolic blood pressure, age and participating hospital.

In the protocol, we stated that the total volume of intracranial haemorrhage would be compared between treatment groups. After publishing the protocol but before unblinding the results,^6^ we proposed (in the Statistical Analysis Plan) that any effect of TXA on intracranial haemorrhage expansion may only be reliably detected in IPH, which is less likely to require urgent neurosurgical evacuation than SDHs and EDHs. Large SDHs and EDHs may require urgent neurosurgical evacuation before we can examine any effect of TXA on the post-randomisation scans. Including these haemorrhages in the primary outcome may dilute any effect of TXA on intracranial haemorrhage expansion to the null.

### Primary outcome analysis plan

A linear mixed model (LMM) was used to compare the mean change in IPH volume from pre-randomisation to post-randomisation between treatment groups.^13^ This model includes pre-randomisation and post-randomisation IPH volumes as correlated outcomes, with the mean post-randomisation IPH volume allowed to differ by treatment group and with the constraint of a common mean pre-randomisation IPH volume across treatment groups.^13^

The advantage of the LMM is that it allows all patients to be included in the analysis, even if they have missing pre-randomisation or missing post-randomisation scans. The IPH volume data from all patients with a pre-randomisation scan and all patients with a post-randomisation scan is included in the analysis. This approach is less biased and more efficient than a model that could only include patients with both pre-randomisation and post-randomisation scans (ie, analysis of covariance).^13^

### Secondary outcomes and analysis plan

*Progressive haemorrhage* is defined as the proportion of patients with a post-randomisation scan with a total bleeding volume (IPH + IVH + EDH + SDH) that is more than 25% of the volume on the pre-randomisation scan. We will examine the effect of TXA on progressive haemorrhage (yes/no) using RRs and 95% CIs. Only patients scanned both pre-randomisation and post-randomisation can be included in this analysis.

*New haemorrhage* is defined as the proportion of patients with any type of bleeding (IPH/IVH/EDH/SDH/SAH) on the post-randomisation scan that was not seen on the pre-randomisation scan. We will examine the effect of TXA on new haemorrhage (yes/no) using RRs and 95% CIs. Only patients scanned both pre-randomisation and post-randomisation can be included in this analysis.

*Cerebral infarction* is defined as the proportion of patients with acute infarction seen on any post-randomisation brain scan done within 28 days of randomisation, excluding patients who had the same infarction on a pre-randomisation scan. All patients with a post-randomisation brain scan on which infarction could be assessed can be included in this analysis, irrespective of whether they were scanned pre-randomisation. We will examine the effect of TXA on cerebral infarction (yes/no) using HRs and 95% CIs. HRs were used (rather than RRs) because TXA may increase cerebral infarction after a more prolonged period after randomisation and the HR accounts for the time from randomisation to the time of the scan on which infarction was seen.

*Composite poor outcome* is defined as the proportion of patients with progressive haemorrhage, new haemorrhage, cerebral infarction or who had neurosurgery or died due to head injury. We will examine the effect of TXA on the composite poor outcome using RRs and 95% CIs. All substudy patients will be included in this analysis.

*Volume of intracranial haemorrhage in patients who undergo surgical evacuation* is defined as the total volume of intracranial haemorrhage (IPH + IVH + EDH + SDH) seen on a scan done post-randomisation and post-neurosurgery in patients who undergo neurosurgical haemorrhage evacuation. A LMM was used to examine the effect of TXA on this outcome.

All secondary outcomes will be adjusted using time from injury to pre-randomisation scan (where appropriate), GCS score, pupil reaction, systolic blood pressure, age and participating hospital.

### Statistical methods

We analysed the data as per the intention-to-treat principle and using the statistical software package Stata (V.15). The analyses involved no statistical imputation. We planned to conduct sensitivity analyses excluding patients who were severely injured at baseline. We assessed the intra-rater and inter-rater reliability of dichotomous outcomes (ie, haemorrhage present/absent) and ordinal outcomes (ie, Marshall classification) using the Kappa (k) statistic, and continuous outcomes (ie, haemorrhage volume) using intraclass correlations (ICC).

## Results

### Study population at baseline

**Figure 2:**
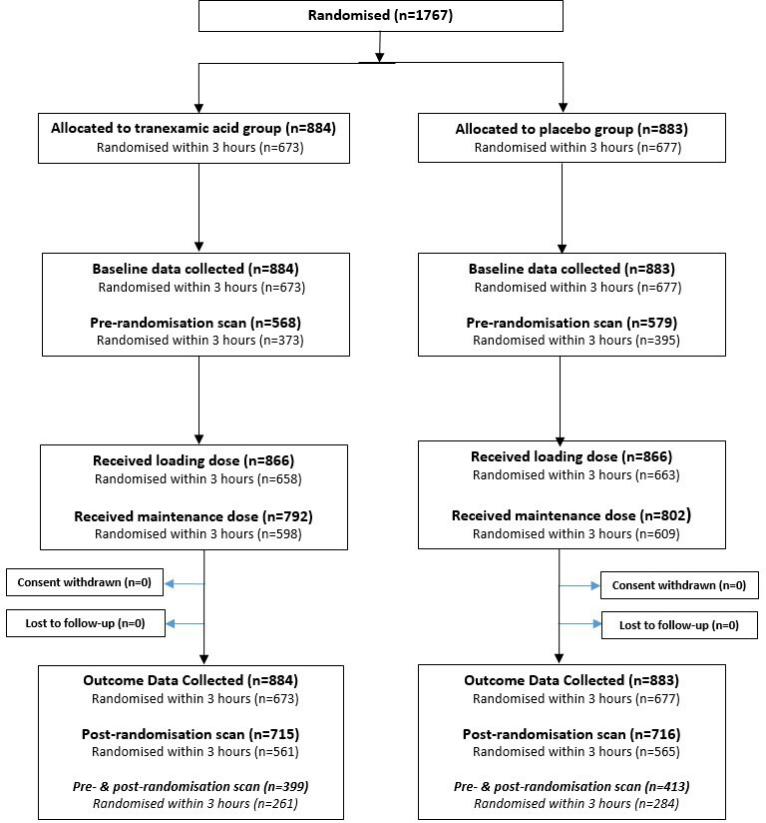
CONSORT (Consolidated Standards of Reporting Trials) diagram on flow of patients in the CRASH-3 trial substudy.

One thousand seven hundred and sixty-seven participants recruited to the CRASH-3 trial between February 2013 and January 2019 were included in this substudy: 884 allocated to TXA group and 883 allocated to placebo group. The CONSORT (Consolidated Standards of Reporting Trials) diagram shows the disposition of patients by treatment group (figure 2) and table 1 shows their pre-randomisation characteristics.

**Table 1:** Baseline demographic and clinical characteristics

	All patients (n=1767)	TXA group (n=884)	Placebo group (n=883)
Sex			
Male	1413 (80%)	701 (79%)	712 (81%)
Female	354 (20%)	183 (21%)	171 (19%)
Age			
Median (IQR) age in years	45 (29 to 63)	45 (29 to 64)	45 (29 to 63)
Glasgow Coma Score (GCS)			
Mild (13 to 15)	92 (5%)	47 (5%)	45 (5%)
Moderate (9 to 12)	532 (30%)	264 (30%)	268 (30%)
Severe (3 to 8)	1143 (65%)	573 (65%)	570 (65%)
Median (IQR) GCS	7 (3 to 10)	7 (3 to 10)	7 (3 to 10)
Pupil reaction			
Both react	1289 (73%)	637 (72%)	652 (74%)
One reacts	202 (11%)	97 (11%)	105 (12%)
None react	232 (13%)	124 (14%)	108 (12%)
Unable to assess	43 (2%)	25 (3%)	18 (2%)
Unknown	1 (<1%)	1 (<1%)	0 (0%)
Systolic blood pressure			
<90	27 (2%)	14 (2%)	13 (1%)
90 to 119	370 (21%)	194 (22%)	176 (20%)
≥120	1362 (77%)	672 (76%)	690 (78%)
Unknown	8 (<1%)	4 (<1%)	4 (<1%)
Median (IQR) systolic blood pressure	136 (120 to 155)	136 (120 to 156)	136 (121 to 154)
Hours since injury			
≤1	166 (9%)	77 (9%)	89 (10%)
>1 to ≤3	1184 (67%)	596 (67%)	588 (67%)
>3	417 (24%)	211 (24%)	206 (23%)
Pre-randomisation CT scan			
Yes	1147 (65%)*	568 (64%)	579 (66%)
No	615 (35%)	313 (35%)	302 (34%)
Unknown	5 (<1%)	3 (<1%)	2 (<1%)
Median (IQR) hours from injury to scan	1.8 (1.4 to 2.4)	1.8 (1.5 to 2.4)	1.8 (1.4 to 2.3)

Data are n (%) of patients, unless otherwise indicated.

*12 unavailable due to technical problems (4 TXA and 8 placebo).

TXA, tranexamic acid.

Sixty-five per cent of the patients (n=1147/1767) had a pre-randomisation CT scan (568 TXA group and 579 placebo group) and 71% of these patients (n=812/1147) also had a post-randomisation scan (399 TXA group and 413 placebo group). Thirty-five per cent were only scanned post-randomisation (n=614/1767) because they had a GCS of ≤12.

The median time to pre-randomisation scan was 2 hours after injury (IQR 1 hour to 2 hours). The median time to the post-randomisation scan was 23 hours after injury (IQR 8 hours to 48 hours).

Of those with a pre-randomisation scan, 97% had intracranial pathology and 82% had intracranial haemorrhage (table 2). The median volume of SDH was 46 mL (IQR 27 to 71 mL), EDH was 6 mL (IQR 2 to 21 mL), IPH was 1 mL (IQR 0.2 to 3.0 mL) and IVH was 0.5 mL (IQR 0.2 to 1.8 mL). The median volume of all haemorrhages combined was 37 mL (IQR 11 to 69 mL).

**Table 2:** Baseline CT scan characteristics

	Patients with baseline CT scan (n=1147)
Intracranial bleeding	
Intraparenchymal	709 (62%)
Intraventricular	184 (16%)
Subdural	732 (64%)
Epidural	215 (19%)
Subarachnoid	829 (72%)
Oedema and infarction	
Oedematous lesions	177 (15%)
Acute cerebral infarction	13 (1%)
Mass effect	
Midline shift	503 (44%)
Median (IQR) degree of shift in mm	6 (4 to 12)
Sulcal effacement	609 (53%)
Ventricular effacement	456 (40%)
Marshall classification	
Diffuse injury I	25 (2%)
Diffuse injury II	533 (46%)
Diffuse injury III	60 (5%)
Diffuse injury IV	30 (3%)
Non-evacuated mass lesion	487 (43%)
Unknown	12 (1%)

Data are n (%) of patients, unless otherwise indicated.

### Primary outcome

IPH volumes were skewed and so medians are presented. The median IPH volume seen after randomisation was 0.5 mL (IQR 0 to 4.6 mL) in the TXA group and 0.3 mL (IQR 0 to 3.1 mL) in the placebo group. Skewed data were transformed into a more normal distribution using log transformation. We used LMM to examine the effect of TXA on log-transformed IPH volumes. Covariates included age, time between injury and the pre-randomisation scan, baseline GCS score and systolic blood pressure, and the participating hospital. Because IPH volumes were log-transformed, we present the anti-log of the effect estimates (and their 95% CIs) to indicate the relative reduction or increase in IPH volume with TXA. In all patients, there was no evidence that TXA prevents IPH expansion: estimate=1.06, 95% CI (0.84 to 1.35), p=0.62. This estimate cannot exclude a reduction or increase in IPH volume with TXA.

### Secondary outcomes

*Progressive haemorrhage*. Among 812 patients scanned pre-randomisation and post-randomisation, 115 (29%) had progressive haemorrhage in the TXA group and 130 (31%) in the placebo group (table 3), adjusted RR=0.91, 95% CI (0.74 to 1.13), p=0.41.

**Table 3:** Effect of TXA on new and progressive haemorrhage

	TXA group	Placebo group	RR (95% CI) *unadjusted*	P value (two-tailed)	RR (95% CI) adjusted*	P value (two-tailed)
New haemorrhage						
≤3 hours since injury	108/261 (41%)	129/284 (45%)	0.91 (0.75 to 1.10)	0.34	0.89 (0.73 to 1.08)	0.22
>3 hours since injury	36/138 (26%)	45/129 (35%)	0.75 (0.52 to 1.08)	0.12	0.69 (0.48 to 0.99)	0.044
All patients	144/399 (36%)	174/413 (42%)	0.86 (0.72 to 1.02)	0.079	0.85 (0.72 to 1.01)	0.069
*Exclude unreactive pupils (one/both*)	108/332 (33%)	140/346 (40%)	0.80 (0.66 to 0.98)	0.033	0.80 (0.66 to 0.98)	0.030
Progressive haemorrhage						
≤3 hours since injury	76/261 (29%)	88/284 (31%)	0.94 (0.73 to 1.22)	0.64	0.92 (0.72 to 1.19)	0.53
>3 hours since injury	39/138 (28%)	42/129 (33%)	0.87 (0.60 to 1.25)	0.45	0.86 (0.58 to 1.28)	0.46
All patients	115/399 (29%)	130/413 (31%)	0.92 (0.74 to 1.13)	0.41	0.91 (0.74 to 1.13)	0.41
*Exclude unreactive pupils (one/both*)	92/332 (28%)	111/346 (32%)	0.86 (0.69 to 1.09)	0.22	0.87 (0.69 to 1.10)	0.24

*Adjusted for time from injury to pre-randomisation scan, age, GlasgowComa Score, pupil reaction, systolic blood pressure and participating hospital.

RR, relative risk; TXA, tranexamic acid.

*New haemorrhage*. Among 812 patients scanned pre-randomisation and post-randomisation, 318 (39%) had a newly detected haemorrhage on the post-randomisation scan: IPH was the most common (58%), followed by IVH (28%), SDH (17%), EDH (6%) and SAH (6%). Fifteen percent of patients with new haemorrhage had more than one type of new haemorrhage. TXA treated patients had fewer new haemorrhages (36% vs 42%: adjusted RR=0.85, 95% CI (0.72 to 1.01), p=0.069). When the 134 patients with unilateral or bilateral unreactive pupils at baseline were excluded, the adjusted RR for a new haemorrhage was 0.80, 95% CI (0.66 to 0.98), p=0.030.

*Cerebral infarction*. Among all 1431 patients with a post-randomisation scan, 11% of patients (n=159/1431) had an acute cerebral infarction, adjusted HR=1.28, 95% (CI (0.93 to 1.76), p=0.13.

*Composite outcome*. There is no evidence for a reduction in the composite with TXA (54% (n=480/884) versus 54% (n=478/883): adjusted RR=0.99, 95% CI (0.91 to 1.07), p=0.83.

*Intracranial haemorrhage in patients who underwent surgical haemorrhage evacuation*. Three hundred and sixty-three patients (21%) underwent neurosurgical haemorrhage evacuation. The median volume of haemorrhage seen after randomisation and neurosurgery was 36 mL (IQR 16 to 61 mL) in the TXA group and 38 mL (IQR 18 to 70 mL) in the placebo group. Haemorrhage volume data were skewed and so log-transformed. We used LMM to examine the effect of TXA on log-transformed haemorrhage volumes. Covariates included age, time between injury and the pre-randomisation scan, baseline GCS score and systolic blood pressure, and the participating hospital. We present the anti-log of the effect estimates and their 95% CIs. There is no evidence that TXA prevents haemorrhage expansion in patients who had neurosurgical haemorrhage evacuation: estimate=0.79, 95% CI (0.57 to 1.11), p=0.63.

### Inter-rater and intra-rater reliability

Inter-rater reliability for pre-randomisation haemorrhage was moderate for most haemorrhage types. EDH ratings agreed between raters in 88% of patients (k=0.57, p<0.0001), IVH in 88% (k=0.50, p<0.0001), SAH in 77% (k=0.50, p<0.0001), SDH in 74% (k=0.43, p<0.0001) and IPH in 65% (k=0.33, p<0.0001). Intra-rater reliability for haemorrhage seen pre-randomisation was fair-to-moderate for most haemorrhage types. IPH ratings agreed in 65% of patients (k=0.32, p=0.0006), SDH in 61% (k=0.33, p<0.0001) and IVH in 94% (k=0.42, p<0.0001). ICCs between individual readings for each patient (ICC=0.70, 95% CI 0.58 to 0.80) and mean readings for each patient (ICC=0.83, 95% CI 0.73 to 0.89) indicate moderate-to-good reliability.

### Missing post-randomisation scans

Three hundred and thirty-five patients did not have post-randomisation scans performed. A larger proportion of patients without (vs with) a post-randomisation scan had a mild GCS score (8% vs 4%: RR=1.87, 95% CI (1.22 to 2.87), p=0.004) or had bilateral unreactive pupils at baseline (22% vs 11%: RR=2.04, 95% CI 1.59 to 2.62, p<0.0001). A larger proportion of patients without (vs with) a post-randomisation scan died from head injury (38% vs 19%: RR=1.97, 95% CI 1.66 to 2.34, p<0.0001) (tables 4 and 5). There was no evidence that TXA increases the risk of not having a post-randomisation scan: 19% (n=169/884) versus 19% (n=166/882), RR=1.02, 95% CI (0.84 to 1.23), p=0.87.

**Table 4:** Patients with post-randomisation scans by baseline injury severity and treatment group

	Patients with post-randomisation scan (n=1431/1767)	TXA group	Placebo group	RR (95% CI)	P value (two-tailed)
Glasgow Coma Score (GCS)					
Mild (GCS 13 to 15)	64 (4%)	32/47 (68%)	32/45 (71%)	0.96 (0.73 to 1.26)	0.75
Moderate (GCS 9 to 12)	425 (30%)	211/264 (80%)	214/267 (80%)	1.00 (0.92 to 1.09)	0.95
Severe (GCS 3 to 8)	942 (66%)	472/573 (82%)	470/570 (82%)	1.00 (0.95 to 1.05)	0.97
Pupil reaction					
Both react	1064 (74%)	529/637 (83%)	535/651 (82%)	1.01 (0.96 to 1.06)	0.68
One reacts	174 (12%)	83/97 (86%)	91/105 (87%)	0.99 (0.88 to 1.10)	0.82
None react	157 (11%)	82/124 (66%)	75/108 (69%)	0.95 (0.80 to 1.14)	0.59
Unable to assess	35 (2%)	20/25 (80%)	15/18 (83%)	0.96 (0.72 to 1.28)	0.78

RR, relative risk; TXA, tranexamic acid.

**Table 5:** Patients without post-randomisation scans by baseline injury severity and treatment group

	Patients without post-randomisation scan (n=335/1767)	TXA group	Placebo group	RR (95% CI)	P value (two-tailed)
Glasgow Coma Score (GCS**)**					
Mild (GCS 13 to 15)	28 (8%)	15/47 (32%)	13/45 (29%)	1.10 (0.59 to 2.06)	0.75
Moderate (GCS 9 to 12)	106 (32%)	53/264 (20%)	53/267 (20%)	1.01 (0.72 to 1.42)	0.95
Severe (GCS 3 to 8)	201 (60%)	101/573 (18%)	100/570 (18%)	1.00 (0.78 to 1.29)	0.97
Pupil reaction					
Both react	224 (67%)	108/637 (17%)	116/651 (18%)	0.95 (0.75 to 1.21)	0.68
One reacts	28 (8%)	14/97 (14%)	14/105 (13%)	1.08 (0.54 to 2.16)	0.82
None react	75 (22%)	42/124 (34%)	33/108 (31%)	1.11 (0.76 to 1.62)	0.59
Unable to assess	8 (2%)	5/25 (20%)	3/18 (17%)	1.20 (0.32 to 4.46)	0.79

RR, relative risk; TXA, tranexamic acid.

Outcomes are presented separately for patients randomised within 3 hours of injury and patients randomised after 3 hours of injury (table 3 and online supplemental appendix 4).

10.1136/emermed-2020-210424.supp4 Supplementary data

## Discussion

### Substudy findings in context of CRASH-3 trial results

The CRASH-3 trial showed that TXA reduces early head injury deaths in non-moribund patients.^2 3^ In this substudy, we attempted to obtain more information about the mechanism of TXA by looking at the brain scans of patients in each trial arm. Performing brain scans was not a requirement of the CRASH-3 trial protocol and so we reviewed clinically indicated scans from a subset of trial patients. Using the date and time of the scans, and the date and time of randomisation into the trial, we were able to confirm whether clinically indicated scans were done before, after, or both before and after randomisation. We did not find evidence for a difference in the volume of intracranial bleeding between TXA and control patients on scans done post-randomisation. Comparing the scans of patients who had both pre-randomisation and post-randomisation scans, we did not find evidence for a reduction in intracranial bleeding with TXA. We did find that the TXA group had fewer new haemorrhages, if we excluded patients whose baseline clinical exam indicated one or both non-reactive pupils.

However, because a large proportion of patients with milder injuries were not scanned after randomisation, we were unable to examine the effect of TXA in the population most likely to benefit. The majority of patients in this substudy had severe head injury and one-third had a GCS score of 3. This was partly because we chose high recruiting hospitals to participate in this substudy, many of which were also major trauma centres, which receive a large proportion of severely injured patients. The CRASH-3 trial found a reduction in head injury death within 24 hours of injury in non-moribund patients.^2 3^ Given the baseline status of the included patients, it would be difficult to see a treatment effect on CT scans in this substudy, which included a large proportion of moribund patients. The baseline data show that patients with severe TBI and unreactive pupils have the largest volumes of intracranial bleeding.^14^ TXA may have little potential to prevent intracranial haemorrhage progression in these patients, and their inclusion may dilute any treatment effect. In fact, much like the CRASH-3 trial which found a reduction in head injury death when patients with unreactive pupils were excluded,^2 3^ when those with unreactive pupils are excluded in this substudy, there is some evidence that TXA prevents new haemorrhage.

### Strengths and weaknesses in relation to other studies

With a limited clinical trial budget, we had to rely on scans that were ordered by treating physicians as part of trial patients’ clinical management. This allowed us to conduct the largest mechanistic study of TXA in TBI to date. However, there are methodological flaws associated with the use of routine imaging. Previous trials in this area were smaller and mandated scans post-randomisation.^9 15 16^ They had fewer missing post-randomisation scans and in this respect are less vulnerable to bias.

### Substudy weaknesses

Although using data collected as part of standard practice in clinical trials can reduce costs, there are threats to validity.^17 18^ In the context of TBI, an initial or repeat CT brain scan may not be performed in patients with mild or unsurvivable injury, and if a second scan is clinically indicated, patients may die before it can be conducted. These missing scans threaten the validity of any inferences. A recent systematic review revealed that there is substantial variability in how missing data are handled in TBI research.^19^ If there is minimal missing data, the impact on effect estimates can be negligible.^20^ In this substudy, one-fifth of the patients did not receive a post-randomisation CT brain scan, which may be related to their prognosis and/or response to TXA.^21^ Because TXA reduces the risk of head injury death,^2^ TXA-treated patients may not have had a clinical indication for a post-randomisation scan and placebo-treated patients may have died before the opportunity for scanning. Therefore, a similar proportion of patients with missing post-randomisation scans in TXA and placebo groups does not exclude the possibility of bias.

One-fifth of the patients had neurosurgical haemorrhage evacuation before the post-randomisation scan. Haemorrhage seen post-randomisation and post-neurosurgery may reflect the combined effects of TXA and neurosurgical haemorrhage evacuation. If TXA reduces intracranial haemorrhage, it may reduce the need for neurosurgical haemorrhage evacuation. Patients who receive placebo and go on to have their intracranial bleed evacuated may have less blood on their post-randomisation scan than those who receive TXA and *do not* undergo evacuation.

The clinical value of an arbitrarily defined progressive haemorrhage outcome is limited. An apparent increase in haemorrhage between scans may not be generalisable because this may have different clinical implications depending on the type of haemorrhage that expands. Even though SDH/EDH are typically larger than IPH/IVH, a 25% increase in SDH/EDH could be managed surgically in the first few hours of injury with good prognostic outcome,^22^ but a 25% increase in IPH/IVH may have worse prognostic outcome.^23^

Outcomes may not have been accurately measured. The ABC/2 method has not been validated in all types of haemorrhage (ie, IVH) and its accuracy is reduced if bleeds are irregularly shaped. SDH volume estimation was based on a novel approach whose reliability is to be confirmed. Unclotted bleeding, micro-bleeding and infarction are not clearly visible on CT, especially soon after injury.^24–26^ Furthermore, reliability ratings indicate that there were discrepancies in ratings between and within assessors. Discrepancies between raters should be expected because the substudy assessor examined scans and radiology reports that are usually written post-randomisation, while randomisation into the CRASH-3 trial was based on information known pre-randomisation (eg, verbal report from clinician). Discrepancies in the substudy assessor’s measurements of the same scans should be expected because Reading 1 was done as part of an audit which primarily involved estimating the volume of the largest bleeds, while Reading 2 was done as part of the substudy procedure and examined all bleeds.

There were two sources of null bias. First, many post-randomisation scans were done very soon after randomisation, when there would have been little opportunity for any effect of TXA to become apparent. This is particularly relevant for types of haemorrhage that usually have smaller volumes, such as IPH, which the baseline data suggest expand at 0.4 mL/hour (IQR 0.1 to 2 mL/hour).^14^ Second, a large proportion of patients had severe (and possibly unsurvivable) head injuries at baseline. Because the CRASH-3 trial did not find evidence for a reduction in head injury death in severely injured patients, their inclusion in this substudy may have also diluted any treatment effect.

### Implications for future research

The US National Institutes of Health propose that ‘the link between prevention of haemorrhage growth (with haemostatic therapy) and clinical outcome’ can be studied using radiological outcomes.^27^ From our experience of conducting this substudy, we believe that this should be done in a large high-quality randomised trial where all randomised patients are scanned post-randomisation. Inclusion should not be based on post-randomisation events or restricted in terms of injury severity. MRI is more sensitive than CT in detecting micro-bleeding^25^ and infarction.^26^

## Conclusion

This substudy was nested within the CRASH-3 trial and its results are consistent with the trial results. Here we found that TXA may prevent new haemorrhage in patients with reactive pupils at baseline, and the CRASH-3 trial found that TXA reduced head injury death in patients with at least one reactive pupil at baseline. While the use of routinely collected scans allowed this substudy to achieve a sample size that is considerably larger than previous studies in this area, this also resulted in many methodological flaws. Most importantly, the large number of patients without post-randomisation scans and the possibility that the availability of scan data depends on whether a patient received TXA, challenges the validity of treatment effect estimates. This study highlights the strengths and limitations of using routinely collected scan data to examine the effects of TBI treatments.
