# Supplementary material for: Effect of tranexamic acid on intracranial haemorrhage and infarction in patients with traumatic brain injury: a pre-planned substudy in a sample of CRASH-3 trial patients
Source: Emerg Med J. 2020 Dec 1;38(4):270–8. doi: 10.1136/emermed-2020-210424 (PMC7982942; doi:10.1136/emermed-2020-210424)
Supplement: Supplementary data [file emermed-2020-210424supp001.pdf]

Appendix 1. Hospitals that participated in the CRASH-3 trial sub-study.

**CRASH-3 Trial Principal Investigator**	**Hospital**	**Number of patients recruited into CRASH-3 trial**	**Number of CRASH-3 trial patients in this study**
Professor Tim Harris	Royal London Hospital (London, UK)	501	314 (63%)
Dr Sabariah Faizah Jamaluddin	Hospital Sungai Buloh (Malaysia)	410	276 (67%)
Professor Antonio Belli	Queen Elizabeth Hospital (Birmingham, UK)	302	267 (88%)
Dr Fatahul Mohamed	Hospital Sultanah Bahiyah (Malaysia)	241	151 (63%)
Dr Caroline Leech	University Hospital Coventry (Coventry, UK)	312	133 (43%)
Dr Mohd Lotfi Hamzah	Hospital Sultanah Nur Zahirah (Malaysia)	205	130 (63%)
Dr Phil Moss	St George's Hospital (London, UK)	280	105 (38%)
Professor Fiona Lecky	Salford Royal Hospital (Salford, UK)	176	72 (41%)
Dr Phillip Hopkins	King's College Hospital (London, UK)	155	64 (41%)
Dr Darin Wong	Penang General Hospital (Malaysia)	161	64 (40%)
Dr Jason Kendall	Southmead Hospital (North Bristol, UK)	156	59 (38%)
Dr Adrian Boyle	Addenbrooke's Hospital (Cambridge, UK)	103	58 (56%)
Professor Mark Wilson	St Mary's Hospital (London, UK)	117	42 (36%)
Dr Melanie Darwent	John Radcliffe Hospital (Oxford, UK)	76	32 (42%)
	Total	*3,195*	*1,767*

Note: 3143 patients were recruited into the CRASH-3 trial in the UK (59 hospitals) and 1567 patients in Malaysia (17 hospitals).
