# Supplementary material for: Effect of tranexamic acid on intracranial haemorrhage and infarction in patients with traumatic brain injury: a pre-planned substudy in a sample of CRASH-3 trial patients
Source: Emerg Med J. 2020 Dec 1;38(4):270–8. doi: 10.1136/emermed-2020-210424 (PMC7982942; doi:10.1136/emermed-2020-210424)
Supplement: Supplementary data [file emermed-2020-210424supp002.pdf]

Appendix 2. Pre-randomisation and post-randomisation CT scan forms (paper version adapted into web version for data collection).

CRASH-3: CT Scan Sub-Study – ISRCTN 15088122

PRE-RANDOMISATION CT SCAN FORM

**CRASH-3 SUB-STUDY: PRE-RANDOMISATION CT SCAN FORM**<br>*Complete as requested and circle where appropriate, please do not leave blanks.*	1. a. ☐ first reader<br>b. ☐ second reader

2.	a.Box					3. Has this patient been scanned before randomisation? YES / NO
	b.Pack					Time between injury and scan (hrs): ______

4. Haemorrhagic findings

a. Is there any intracranial bleeding on CT scan? *(circle one option on each line)*	YES	NO	*If NO, go to Question 5*<br>*If YES, continue*							
**Please specify type and volume of haemorrhage:**										
b. Parenchymal	YES	NO								
bii. Code	A	B	C	(ABC/2)/1000	HU*	Note				
c. Subdural	YES	NO	*(measure width only**)*							
cii. Code		B			HU	Note				
d. Epidural	YES	NO								
dii. code	A	B	C	(ABC/2)/1000	HU	Note				
e. Intraventricular	YES	NO								
eii. code	A	B	C	(ABC/2)/1000	HU	Note				
f. Petechial	YES	NO	Note:							
g. Subarachnoid	YES	NO	*(tick one option per line for each haemorrhage)*							
gii. code	Small			Medium			Large			HU
	F	M	D	F	M	D	F	M	D	

Codes

L: Left
R: Right

T: Temporal
F: Frontal
P: Parietal
O: Occipital

BG: Basal Ganglia
B: Brainstem

Abbreviations

A: maximal diameter (mm);
B: maximal diameter perpendicular to A (mm);
C: number of slices on which haemorrhage is visible multiplied by slice thickness (mm);

HU: Hounsfield Unit

F: Focal
M: Multiple
D: Diffuse

5. CT characteristics

Marshall Classification *(circle YES to the most severe option only)*		
a. Diffuse injury I (no visible pathology)	YES	NO
b. Diffuse injury II (cisterns present with midline shift 0-5mm; no lesion >25cm^3)	YES	NO
c. Diffuse injury III (cisterns compressed/absent with midline shift 0-5mm; no lesion > 25cm^3)	YES	NO
d. Diffuse injury IV (midline shift > 5mm; no lesion > 25cm^3)	YES	NO
e. Evacuated mass lesion (any lesion surgically evacuated)	YES	NO
f. Non-evacuated mass lesion (lesion >25cm^3; not surgically evacuated)	YES	NO

6. Non-haemorrhagic findings *(circle one option on each line)*

a. Is there any sign of acute focal ischaemic lesion?	YES	NO	Volume:	A: ___ mm	B: ___ mm	C: ___ mm	ABC/2: ___ cm^3
b. Are there any oedematous lesions?	YES	NO	bi. *total volume in ml*				

7. Mass effect findings *(circle one option on each line)*

Please specify if any of the following ***mass effect*** signs are present:				
a. Sulcal effacement	YES	NO		
b. Ventricular effacement	YES	NO		
c. Midline shift	YES	NO	ci. *approximate shift (mm)*	
**If yes** to any of the above, is the mass effect caused by:				
d. Haemorrhage	YES	NO		
e. Oedema	YES	NO		
f. Both	YES	NO		

8. Neurosurgery

a. Did the patient undergo neurosurgery?	YES	NO	b. Type of surgery: ______________ ______________	c. Date of surgery	___/___/____ (dd/mm/yyyy)

9. Details of reading

a. Name of the person completing the form	______________	b. Date of reading	___/___/____ (dd/mm/yyyy)

Has this patient been scanned after randomisation into the trial?	YES / NO
Any relevant patient notes ______________	

*Place curser on most visually dense portion of lesion
**If the width of a subdural bleed is greater than 6mm, we can assume the volume is 27cm^3 (i.e. to aid rating the Marshall Classification)

CRASH-3: CT Scan sub-study – ISRCTN 15088122

POST-RANDOMISATION CT SCAN FORM

**CRASH-3 SUB-STUDY: POST-RANDOMISATION CT SCAN FORM**<br>*Complete as requested and circle where appropriate, please do not leave blanks.*						1. a. ☐ first reader<br>b. ☐ second reader
2.	a.Box					3. Time between randomisation and scan (hrs): _______
	b.Pack					
Are the follow-up CT scan parameters comparable with the initial CT scan?					YES	NO

4. Haemorrhagic findings

a. Is there any intracranial bleeding on CT scan?<br>*(circle one option on each line)*	YES	NO	*If NO, go to Question 5*<br>*If YES, continue*			
**Please specify type and volume of haemorrhage:**						
b. Parenchymal	YES	NO				
bii. Code	A	B	C	(ABC/2)/1000)	HU*	Note
c. Subdural	YES	NO	*(measure width only**)*			
cii. Code		B			HU	Note
d. Epidural	YES	NO				
dii. code	A	B	C	(ABC/2)/1000	HU	Note
e. Intraventricular	YES	NO				
eii. code	A	B	C	(ABC/2)/1000	HU	Note
f. Petechial	YES	NO	Note:			
g. Subarachnoid	YES	NO	*(tick one option per line for each haemorrhage)*			

gii. code	Small			Medium			Large			HU
	F	M	D	F	M	D	F	M	D	
h. Is this a new haemorrhage (not seen on pre-randomisation scan)?									YES	NO
hi. Give details (e.g. subdural present on pre-randomisation but not follow up):										

Codes

L: Left
R: Right

T: Temporal
F: Frontal
P: Parietal
O: Occipital

BG: Basal Ganglia
B: Brainstem

Abbreviations

A: maximal diameter (mm);
B: maximal diameter perpendicular to A (mm);
C: number of slices on which haemorrhage is visible multiplied by slice thickness (mm);

HU: Hounsfield Unit

F: Focal
M: Multiple
D: Diffuse

5. CT characteristics

**Marshall Classification** *(circle YES to the most severe option only)*		
a. Diffuse injury I (no visible pathology)	YES	NO
b. Diffuse injury II (cisterns present with midline shift 0-5mm; no lesion >25cm^3)	YES	NO
c. Diffuse injury III (cisterns compressed/absent with midline shift 0-5mm; no lesion > 25cm^3)	YES	NO
d. Diffuse injury IV (midline shift > 5mm; no lesion > 25cm^3)	YES	NO
e. Evacuated mass lesion (any lesion surgically evacuated)	YES	NO
f. Non-evacuated mass lesion (lesion >25cm^3; not surgically evacuated)	YES	NO

6. Non-haemorrhagic findings *(circle one option on each line)*

a. Is there any sign of acute focal ischaemic lesion?	YES	NO	Volume:	A: ___ mm	B: ___ mm	C: ___ mm	ABC/2: _____ cm^3
ai. Is this a new acute focal ischaemic lesion (not seen on the pre-randomisation scan)?	YES	NO					
b. Are there any oedematous lesions?	YES	NO	bi.*total volume in ml*				

7. Mass effect findings *(circle one option on each line)*

Please specify if any of the following ***mass effect*** signs are present:				
a. Sulcal effacement	YES	NO		
b. Ventricular effacement	YES	NO		
c. Midline shift	YES	NO	ci. *approximate shift in mm*	
If yes to any of the above, is the mass effect caused by:				
d. Haemorrhage	YES	NO		
e. Oedema	YES	NO		
f. Both	YES	NO		

8. Neurosurgery

a. Did the patient undergo neurosurgery?	YES	NO	b. Type of surgery: ____________________ ____________________	c. Hours between surgery and CT scan:_______

9. Details of reading

a. Name of the person completing the form	______________________________	c. Date of reading	____/____/____ (dd/mm/yyyy)

10. Ischaemic lesions on further post-randomisation scans

ai. Is there a second post-randomisation scan?	YES	NO
aii. If there is a second post-randomisation scan, is there a new focal ischaemic lesion?	YES	NO
bi. Is there a third post-randomisation scan?	YES	NO
bii. If there is a third post-randomisation scan, is there a new focal ischaemic lesion?	YES	NO

Any relevant patient notes (including info on further scans)	______________________________
