# Supplementary material for: Effect of tranexamic acid on intracranial haemorrhage and infarction in patients with traumatic brain injury: a pre-planned substudy in a sample of CRASH-3 trial patients
Source: Emerg Med J. 2020 Dec 1;38(4):270–8. doi: 10.1136/emermed-2020-210424 (PMC7982942; doi:10.1136/emermed-2020-210424)
Supplement: Supplementary data [file emermed-2020-210424supp003.pdf]

Appendix 3. Formula to estimate subdural haemorhage volume

$$\frac{\left[4/3\ \pi\ (6.85)^3 - 4/3\ \pi\ (6.85\text{-}r)^3\right]}{8}$$

We used the longitudinal diameter (temporal-temporal): 137mm or 13.7cm (6.85cm radius).

r is the maximum thickness of the subdural haemorrhage.

For example, for a subdural haemorrhage with a thickness of **7.7mm** (i.e. 0.77cm):
$4/3\pi(6.85)^3 =$ 1346.35728

$4/3\pi(6.85 - \mathbf{0.77})^3 = 4/3\pi(6.08)^3 = 941.45452$

1346.35728 - 941.45452 = 404.90276

404.90276 / 8 = 51cm^3 (i.e. **51ml**)

For example, for a subdural haemorrhage with a thickness of **3.5mm** (i.e. 0.35cm):
$4/3\pi(6.85 - \mathbf{0.35})^3 = 4/3\pi(6.5)^3 = 1150$

1346 – 1150 = 196

196 / 8 = **25ml**

For example, for a subdural haemorrhage with a thickness of **6mm** (i.e. 0.6cm):
$4/3\pi(6.85 - \mathbf{0.6})^3 = 4/3\pi(6.25)^3 = 1023$

1346 - 1023 = 323

323 / 8 = **40 ml**
