# Supplementary material for: Effect of tranexamic acid on intracranial haemorrhage and infarction in patients with traumatic brain injury: a pre-planned substudy in a sample of CRASH-3 trial patients
Source: Emerg Med J. 2020 Dec 1;38(4):270–8. doi: 10.1136/emermed-2020-210424 (PMC7982942; doi:10.1136/emermed-2020-210424)
Supplement: Supplementary data [file emermed-2020-210424supp004.pdf]

Appendix 4. Outcomes in patients randomised within/after three hours of injury.

Intracranial haemorrhage

There is no evidence that TXA prevents IPH expansion in patients randomised within 3 hours of injury (1.09, 95% CI (0.81–1.45), p=0.570) or after three hours of injury (0.95, 95% CI (0.63–1.43), p=0.789). There is no evidence that TXA prevents haemorrhage expansion in patients who had neurosurgical haemorrhage evacuation and were randomised within 3 hours of injury (n=277) (0.94, 95% CI (0.62–1.42), p=0.756) but there is some evidence in those randomised after three hours of injury (n=86) (0.37, 95% CI (0.19–0.72), p=0.003).

Cerebral infarction

There is no evidence for an increase in infarction with TXA at any particular time post-randomisation in those randomised within 3 hours of injury (adjusted HR=1.21, 95% CI (0.85–1.73), p=0.297) or after 3 hours of injury (adjusted HR=1.68, 95% CI (0.78–3.59), p=0.185).
